# Is it Worth the Effort? Novel Insights into Obesity-Associated Alterations in Cost-Benefit Decision-Making

**DOI:** 10.3389/fnbeh.2015.00360

**Published:** 2016-01-12

**Authors:** David Mathar, Annette Horstmann, Burkhard Pleger, Arno Villringer, Jane Neumann

**Affiliations:** ^1^Department of Neurology, Max Planck Institute for Human Cognitive and Brain SciencesLeipzig, Germany; ^2^IFB Adiposity Diseases, Leipzig University Medical CenterLeipzig, Germany; ^3^Clinic of Cognitive Neurology, University Hospital LeipzigLeipzig, Germany; ^4^Berlin School of Mind and Brain, Mind and Brain Institute, Humboldt-UniversityBerlin, Germany

**Keywords:** obesity, cost-benefit decision-making, physical effort, reward, voxel-based morphometry

## Abstract

Cost-benefit decision-making entails the process of evaluating potential actions according to the trade-off between the expected reward (benefit) and the anticipated effort (costs). Recent research revealed that dopaminergic transmission within the fronto-striatal circuitry strongly modulates cost-benefit decision-making. Alterations within the dopaminergic fronto-striatal system have been associated with obesity, but little is known about cost-benefit decision-making differences in obese compared with lean individuals. With a newly developed experimental task we investigate obesity-associated alterations in cost-benefit decision-making, utilizing physical effort by handgrip-force exertion and both food and non-food rewards. We relate our behavioral findings to alterations in local gray matter volume assessed by structural MRI. Obese compared with lean subjects were less willing to engage in physical effort in particular for high-caloric sweet snack food. Further, self-reported body dissatisfaction negatively correlated with the willingness to invest effort for sweet snacks in obese men. On a structural level, obesity was associated with reductions in gray matter volume in bilateral prefrontal cortex. Nucleus accumbens volume positively correlated with task induced implicit food craving. Our results challenge the common notion that obese individuals are willing to work harder to obtain high-caloric food and emphasize the need for further exploration of the underlying neural mechanisms regarding cost-benefit decision-making differences in obesity.

## Introduction

Everyday decisions are guided by cost-benefit analyses. For example, whether or not we choose to walk to the next store to buy chocolate depends not only on how much we like chocolate and on our present state of hunger, but also on our subjective perception of the distance to the next store. Thus, we weigh the expected rewards an action will deliver against the effort for obtaining them to calculate a subjective utility value that guides our decisions.

Several studies have revealed that perceived effort strongly impacts on our decisions and that the fronto-striatal pathway is critical for integrating effort costs to form a decision in both animals and humans (Salamone et al., 1994; Bautista et al., 2001; Walton et al., 2002, 2006; Croxson et al., 2009; Basten et al., 2010). Mesolimbic dopamine was shown to play a key role in the motivation to overcome costs in order to receive rewards (Salamone et al., 1994, 2007; Kurniawan et al., 2011; Salamone and Correa, 2012; Treadway et al., 2012). In particular nucleus accumbens (NAcc) dopamine is believed to modulate motivational salience in goal-directed behavior (Salamone and Correa, 2012).

In the case of obesity, identifying possible alterations in cost-benefit decision-making is of particular importance. In our obesogenic environment potent food is always available at minimal costs, and excess weight is associated with a reduced motivation for physical activity (Ness et al., 2007) and a possibly heightened valuation of potent food rewards (e.g., Rothemund et al., 2007).

Obesity has been characterized by a reduced binding potential of striatal dopamine receptors (Wang et al., 2001; de Weijer et al., 2011). This is hypothesized to be associated with a heightened striatal dopaminergic tone (Horstmann et al., 2015b). Further, obese individuals show a heightened neural activation during the anticipation of high-caloric food stimuli in dopaminergic target regions such as NAcc in the context of reward processing (e.g., Stice et al., 2008; Nummenmaa et al., 2012). This food-related hyper-responsiveness may point at a context-sensitive dopaminergic reward system, possibly mediated through food-related memory input from the ventral subiculum of the hippocampus (Belujon and Grace, 2011).

To date, only few studies have explored obesity-associated alterations in cost-benefit decision-making in humans. Two studies indicate that obese subjects may be willing to invest more effort to obtain high-caloric food than lean individuals (Epstein et al., 2007; Giesen et al., 2010). Both studies used button presses as a measure of physical effort and assessed obesity-associated alterations in cost-benefit decisions solely with respect to food reward. It is thus not clear if the results generalize to other reward categories. Further, findings in rodents related to cost-benefit decision-making in obesity are mixed, with some studies showing a heightened willingness to work for food rewards in diet-induced obese animals (e.g., Narayanaswami et al., 2013) and others revealing the opposite (e.g., Harb and Almeida, 2014).

Thus, there is need for a systematic investigation of cost-benefit decision-making in obesity that involves properly demanding physical effort measures and food and non-food reward categories.

Importantly, cost-benefit decision-making, with its strong reliance on dopaminergic pathways, may be modulated by a multitude of factors, in addition to obesity. Hence, a thorough investigation of obesity-associated cost-benefit decision making necessitates the inclusion of possible confounding factors. Gender has been revealed to alter dopaminergic neurotransmission, and recently estradiol was shown to modulate dopamine baseline dependent cognitive functioning in humans (Haaxma et al., 2007; Jacobs and D'Esposito, 2011) and cost-benefit decision-making in rodents (Uban et al., 2012). Further, gender has been shown to modulate obesity-related differences in brain structure and related processes of cognitive control (Horstmann et al., 2011). In the context of reinforcement sensitivity theory, reward and punishment sensitivity are both known to rely on dopaminergic activity (Maril et al., 2013; Tomer et al., 2014) and may impact sensitivity to rewards and effort demands in cost-benefit decisions. Further, both are associated with eating behavior (Loxton and Dawe, 2006; Matton et al., 2013; Dietrich et al., 2014). In the case of obesity, additional psychosocial factors, such as concerns about one's own body image, may modulate food-related cost-benefit decisions. Following findings regarding stigmata in obesity (e.g., Forste and Moore, 2012), one would for example expect self-reported body dissatisfaction to decrease motivation to invest effort for high-caloric food specifically in obese women. As a potent environmental factor affecting effort-based decision-making, stress was shown to modulate dopaminergic transmission within the fronto-striatal system (Nagano-Saito et al., 2013; Pruessner et al., 2013) and to reduce intrinsic motivation to invest effort in rodents (Shafiei et al., 2012). Stress has also been linked to eating behavior and body weight (Warne, 2009; Tomiyama et al., 2011).

In this study we investigate obesity-associated alterations in cost-benefit decision-making and in related reaction times regarding physical effort and different kinds of food and non-food rewards. We designed a novel cost-benefit decision-making paradigm in which subjects can choose to invest physical effort via a digital handgrip device to receive rewards out of three distinct categories: money, fruit and sweet high-caloric snacks. We hypothesize that obese compared with lean subjects show alterations in cost-benefit decision-making particularly in relation to high-caloric sweet snack food. We expect that gender and subjects' self-reported body dissatisfaction strongly modulate willingness to exert effort for sweet snacks in obese participants. Obese participants with high body dissatisfaction may be less driven to invest effort for high-caloric food reward compared with lean subjects. The impact of body dissatisfaction on subjects' cost-benefit decisions may be more prominent in women than in men. Further, we expect that perceived chronic stress level negatively correlates with subjects' motivation to invest effort. As physical effort may be experienced as a sort of punishment, high punishment sensitivity may reduce willingness to exert effort, whereas heightened reward sensitivity may enhance willingness to invest effort to receive rewards.

Beyond behavioral assessment, we apply voxel-based morphometry (VBM) in a subsample of our subjects. Based on recent findings (Schäfer et al., 2010; Horstmann et al., 2011), we hypothesize that obese compared with lean subjects show lower gray matter volume in cognitive-control related lateral prefrontal cortices (lPFC) and possibly higher gray matter volume in areas implicated in reward processing such as orbitofrontal cortex (OFC) and NAcc. We expect that volume of NAcc, as the dopaminergic core brain structure involved in the motivation to overcome costs to obtain rewards, positively correlates with subjects' willingness to exert effort. In addition, as NAcc activation has been related to craving severity (Kober et al., 2010) and its gray matter volume to eating behavior pathology and addiction-like behavior (Schäfer et al., 2010; Howell et al., 2013), we predict that NAcc gray matter volume positively correlates with task induced craving for high-caloric food.

## Materials and methods

### Subjects

The study was carried out in compliance with the Declaration of Helsinki and approved by the local ethics committee of the University of Leipzig. We included 57 healthy Caucasian participants who were separated into four groups according to their BMI and gender: two obese (BMI ≥ 30, BMI < 40) groups with 14 female and 15 male participants, and two lean control groups (BMI ≥ 19, BMI ≤ 25), consisting of 15 female and 13 male subjects. The four groups were closely matched for educational background (i.e., years of scholastic education) and age distribution (Table 1). All participants were right-handed (Edinburgh Handedness Inventory, (Oldfield, 1971)), between 18 and 35 years old and reported to generally like fruits and sweets. Exclusion criteria were hypertension, dyslipidemia, metabolic syndrome, depression [Beck Depressions Inventar (BDI), Hautzinger, 1995 (german version of the Beck Depression Inventory (BDI-II), Beck et al., 1996; cut-off value 18; Table 1], a history of neuropsychiatric diseases, smoking, and diabetes mellitus type I and II. All subjects gave written informed consent before taking part in the study.

**Table 1:** **Sample size, distribution of body mass index (BMI), age, years of education, depressive symptoms (BDI), punishment sensitivity (BIS), reward sensitivity (BAS), chronic stress level (TICS), self-reported body dissatisfaction, VAS hunger rating prior to experiment, task-induced implicit food craving, nine-point Likert Scale rating of subjects' wanting and liking of the individual sweet snack and fruit items that entered the task, subjects' maximum hand grip force, average reaction times and fraction of choices to exert effort throughout the task**.

	**Lean women**	**Obese women**	**Lean men**	**Obese men**	***F*-/*H*-values**	***p***
Sample size (sample size MRI)	15(12)	14(8)	13(11)	15(11)	–	–
BMI	22.1±1.3	33.6±2.0	21.4±1.3	33.5±2.6	*F*_(3, 53)_ = 182.11	<**0.001**
Age	24.3±3.0	26.5±4.5	26.1±3.0	27.5±3.6	*F*_(3, 53)_ = 2.05	0.12
Years of education	13(13–13)	13(10–13)	13(13–13)	13(10–13)	*H*_(3)_ = 3.89	0.27
BDI	2.7±2.9	6.1±5.1	3.5±4.0	4.5±4.5	*H*_(3)_ = 3.90	0.27
BIS	19.2±2.5	18.8±2.7	18.4±3.3	17.7±3.7	*F*_(3, 53)_ = 0.67	0.58
BAS	38.3±9.3	37.9±8.1	41.1±4.9	37.6±9.0	*F*_(3, 53)_ = 0.48	0.70
TICS	16.8±7.5	18.4±9.2	15.2±7.1	16.9±9.7	*F*_(3, 53)_ = 0.30	0.82
Body dissatisfaction	29.8±10.3	44.1±8.8	16.0±5.3	38.5±10.8	*F*_(3, 53)_ = 22.62	<**0.001**
Hunger prior to experiment	59.4±22.7	56.2±28.1	53.3±20.7	58.6±19.5	*F*_(3, 53)_ = 0.02	0.89
Implicit food craving	22.7±18.3	32.4±25.3	21.7±20.3	17.6±15.8	*F*_(3, 53)_ = 1.43	0.24
Wanting of included sweet items	7.7±1.1	7.2±1.0	7.5±1.0	6.7±1.4	*H*_(3)_ = 5.82	0.12
Wanting of included fruit items	8.1±0.8	7.6±1.1	7.9±1.0	7.8±0.8	*H*_(3)_ = 2.39	0.50
Liking of included sweet items	8.1±0.9	7.3±0.8	7.5±0.9	7.1±0.9	*H*_(3)_ = 8.13	<**0.05**
Liking of included fruit items	8.3±0.7	7.8±0.8	8.1±1.0	8.2±0.8	*H*_(3)_ = 4.33	0.23
Maximum grip force	27.1±5.5	27.1±5.6	43.1±6.9	48.1±9.2	*F*_(3, 53)_ = 34.96	<**0.001**
Reaction times	581.9±74.1	672.3±106.6	569.1±62.9	614.2±105.8	*F*_(3, 53)_ = 3.63	<**0.05**
% Choices of effort exertion	64.4±15.0	65.4±18.7	71.4±17.0	56.6±11.1	*F*_(3, 53)_ = 2.17	0.10

*Values represent mean ± standard deviation, except for years of education [median (min max)]. Tests for group differences are based on Kruskal–Wallis–H-tests and ANOVA (F). Bold values represent significant group differences*.

### Stimuli

In our experiment, subjects could earn rewards from three distinct categories: money, fruit, and sweet snacks, and in two quantities: one or four pieces. Money was represented by two cent coins. Available fruit items included pieces of apple, banana, kiwi, nectarine, orange, pear, physalis, pineapple, raisins, and strawberries. Sweet snack items consisted of different small chocolate bars from Mars' celebration collection (Bounty, Dove, Dove-Caramel, Mars, Milkyway, Snickers, Teaser, Twix) and four different gummi bear-like snacks (Haribo Goldbären, Haribo Konfekt, Haribo Vampire, and Saure Apfelringe). During the task, reward stimuli were presented as photographs of the respective reward item that showed either one or four pieces, indicating the quantity of the reward item subjects could earn in the respective trial.

To earn the rewards during the task, subjects had to exert handgrip force. Force levels consisted of an easy and a hard category, with force levels drawn from two normal distributions with mean 50 or 67% and standard deviation 2% of subjects' individual maximum handgrip forces. A thermometer on the screen indicated the proposed level.

Before being instructed, subjects' maximum handgrip force was assessed with an isometric handgrip device (BIOPAC, TSD-121) to individually adjust effort levels in the subsequent task. To familiarize participants with the task, they performed 10 practice trials beforehand. During the task, subjects made their choices with a response pad in their left hand and exerted effort with the isometric handgrip device in their right hand. Subjects' right hand was videotaped to assure that subjects only gripped with their right hand during the task.

### Visual analog scale (VAS) rating and questionnaires

Subjects were told to refrain from eating 3 h before the experiment when they were invited. When they entered the lab, subjects completed several questionnaires related to body dissatisfaction (EDI-2, Paul and Thiel, 2005; Table 1), reward and punishment sensitivity (German version of BIS/BAS scale, Carver and White, 1994; Strobel et al., 2001; Table 1), and chronic stress levels (TICS, Schulz et al., 2004; Table 1). Prior to performing the task, subjects were asked to rate how hungry they felt on a VAS (range: 0–100; 0, not hungry at all; 100, extremely hungry), to control for differences in hunger. After the task subjects were asked to rate their state of hunger again. We used the hunger difference before and after the task as an implicit measure of task-induced food craving.

### Food item rating

To control for individual liking and wanting of the food items that entered the task, we assessed subjects' liking and wanting of the food items. Specifically, subjects were presented pictures of all food items on the computer screen in a randomized order and asked to rate them according to how much they liked the respective food. Subsequently, subjects were asked to rate the food items with respect to how much they wanted to eat the different food items right now. Liking and wanting ratings were obtained utilizing nine-point Likert scales, ranging from “not at all” (1) to “very much” (9). For each subject, the five “most wanted” sweet snacks and fruit items were chosen as stimuli in the subsequent cost-benefit task.

### Task

**Figure 1:**
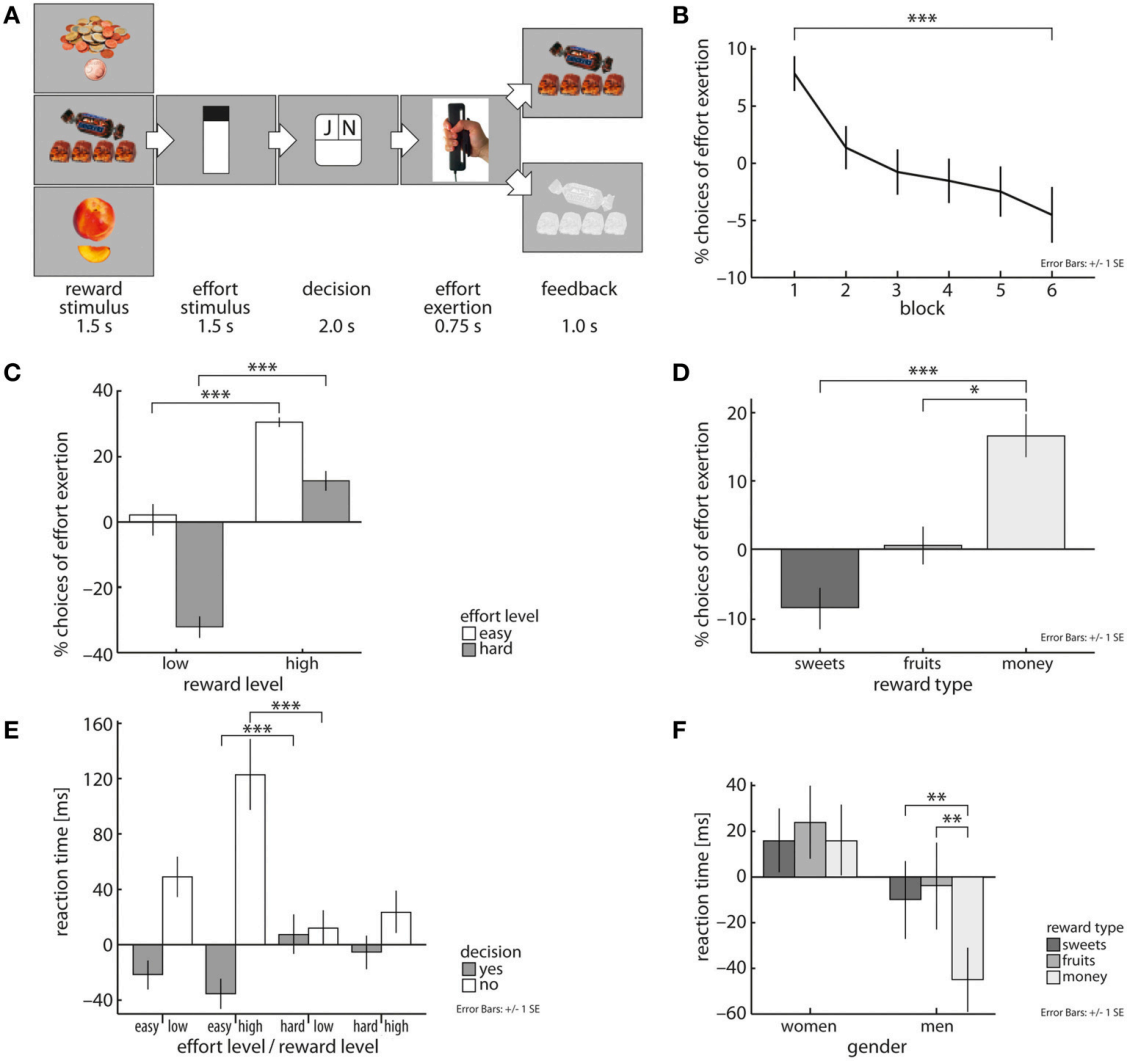
Schematic representation of the novel cost-benefit decision-making task **(A)**. Across all subjects, likelihood of choosing to grip decreases over task blocks (40 trials each) **(B)** and is dependent on both effort and reward magnitude **(C)**. Subjects exerted effort more often for money than for fruit and sweet snacks **(D)**. Subjects decided fastest to expend effort in trials with low effort and high reward magnitudes and decided slowest to reject these offers **(E)**. Men decided faster in trials involving monetary reward than in food reward trials **(F)**. Depicted values are corrected for factors and covariates within the respective GEE model. Asterisks indicate significance within the respective GEE model reported in the Results Section.

In each trial of the task (Figure 1A), subjects were shown a picture of the available reward item (one or four pieces of the sweet snack, fruit, or money) and the required effort level they had to invest (high or low, indicated by a thermometer). In half of the trials, the order of reward and effort presentation was reversed. Subsequently, subjects decided whether they wanted to exert the effort level to receive the reward item or not. Subjects were instructed to decide as fast as possible. Reward and effort presentation each lasted for 1500 ms followed by a 2000 ms time interval where subjects had to indicate on a two button response pad in their left hand, whether they wanted to exert the respective effort level to receive the anticipated reward, or not. If they did not respond in time, a frowney appeared on the screen together with the instruction “too slow.” Assignment of “yes” and “no” to the two response buttons was randomized over trials. After the decision, subjects either had to grip the required force level or passively waited for 2000 ms. If they chose to grip, subjects had to squeeze the hand grip device until the indicated level was reached on the thermometer within 2000 ms and had to maintain this force level for 750 ms. If subjects successfully finished the effort exertion phase, they received a positive feedback that consisted of a smiley and the earned reward displayed for 1000 ms. If they failed to do so or had chosen not to grip, they received negative feedback in the form of a frowney and a masked picture of the reward. The next trial started after an inter-trial-interval of 2000 ms. Reward category, reward and effort level and trial order (reward first/effort first) were randomized over the whole task and each block.

The task consisted of six blocks (lasting ~10 min) composed of 40 trials each, leading to a total of 240 trials. Between trial blocks, subjects had 3 min time to relax their right hand from gripping. At the end of the task, subjects' maximum grip force was assessed again, to exclude that cost-benefit decisions were influenced by fatigue. Finally, subjects were paid a compensation of 7 €/h and received their earned sweet snacks, fruit pieces and additional reimbursement of up to 3 € according to their gained reward across money trials.

### MRI acquisition

We acquired T1-weighted images in a subsample of 42 [20 female, 19 (8 female) obese] participants on a whole-body 3T TIM Trio scanner (Siemens, Erlangen, Germany) with a 12-channel head-array coil using a MPRAGE sequence [TI = 650 ms; TR = 1300 ms; snapshot FLASH, TRA = 10 ms; TE = 3.93 ms; α = 10°; bandwidth = 130 Hz/pixel (i.e., 67 kHz total); image matrix = 256 × 240; FOV = 256 × 240 mm; slab thickness = 192 mm; 128 partitions; 95% slice resolution; sagittal orientation; spatial resolution = 1 × 1 × 1.5 mm; two acquisitions].

### Image processing

Image pre-processing and statistical analysis were performed using SPM8 (Wellcome Trust Centre for Neuroimaging, UCL, London, UK; http://www.fil.ion.ucl.ac.uk/spm) running under MatLab 7.14 (Mathworks, Sherborn, MA, USA). MR images were processed using Diffeomorphic Anatomical RegisTration using Exponentiated Lie algebra (DARTEL) (Ashburner, 2007) with standard parameters for VBM. All analyses were performed on bias-corrected, segmented, registered (rigid-body transformation), interpolated isotropic (1.5 × 1.5 × 1.5 mm), and smoothed (FWHM 8 mm) images. All images were warped based on the transformation of the standard MNI152 DARTEL template to the GM prior image provided by SPM8 to meet the standard stereotactical space of the Montreal Neurological Institute (MNI). GM segments were non-linearly scaled by the Jacobian determinants of the deformations introduced by normalization to account for local compression and expansion during transformation.

### Data analysis

#### Behavioral data

All analyses were performed with PASW-SPSS-Statistics 22.0 (IBM Corporation, Somers, NY, USA). A possible group-related difference with respect to subjects' VAS ratings of hunger was assessed by ANOVA. Differences in nine-point Likert scale liking and wanting ratings of individual food reward items that entered the task were assessed by Mann-Whitney U-tests. *Post-hoc* pair-wise comparisons were computed with two sample *T*-tests or Mann-Whitney U-tests and adjusted for pairwise comparisons.

We applied a generalized estimating equations (GEE) approach to assess the impact of our variables of interest on subjects' reaction times and on subjects' single trial binary decisions. GEE is an extension of a generalized linear model that is capable of accounting for possible unknown correlations between residuals. An advantage of GEE is that its parameter estimates are consistent under mild regularity conditions, even when the covariance structure is mis-specified (Zeger and Liang, 1986). Computed GEE models utilized a first order autoregressive working correlation matrix, a linear normal outcome distribution for modeling reaction times and a binary logistic outcome distribution to model subjects' decisions to grip or not on each single trial, respectively.

We computed four successive GEE models for both reaction times and choice analysis, respectively. The first model contained as predictor variables trial block, trial order (reward first/effort first), reward category, reward and effort magnitude, and the interaction of the two latter. Based on our hypotheses we included obesity, gender, subjects' reward and punishment sensitivity, and perceived chronic stress as further predictors. To control for individual differences in wanting and possible effects of age, we included subjects' wanting ratings of the food items, and age as covariates. For analysis purposes, we set wanting ratings constantly to the highest value (9) for monetary reward, assuming that every participant wanted to receive money. As we wished to assess whether reaction times differed with respect to subjects' choices (yes/no), we also included the binary decision for each single trial and a three-way interaction term of effort magnitude, reward magnitude, and decision into the first GEE for reaction time analysis.

Covariates that yielded no statistical significance in the first GEE model, at least on a trend level (*p* < 0.1), were disregarded in subsequent GEE models. In the second GEE, we tested for obesity- and gender-specific two-way interactions, i.e., gender by effort magnitude, gender by reward category, obesity by effort level, obesity by reward category, and obesity by gender. The second model for choice analysis also included the interactions of obesity by trial block and gender by trial block to assess group-related differences over time. According to our hypotheses, the third GEE was set up to assess a three-way interaction of reward category, obesity, and gender. To test for a modulatory effect of body dissatisfaction, we assessed a four-way interaction between reward category, obesity, gender, and body dissatisfaction with the last GEE model.

Age, reward sensitivity, punishment sensitivity, chronic stress, and individual wanting ratings were mean centered prior to analyses. Self-reported body dissatisfaction was centered on the respective group mean with regard to gender and obesity, as we were interested in its impact specifically for each group.

*Post-hoc* analyses of interaction effects were based on estimated marginal means (EMMs) of the mean response for reaction time analyses and on EMMs of the linear predictor of the binary outcome variable “decision” and consisted of Wald Chi-Square Tests and ANOVAs for pairwise comparisons, corrected for multiple comparisons utilizing Bonferroni correction.

#### Structural MRI data

We utilized a full-factorial design within SPM8 running under Matlab 7.14 with factors obesity and gender, and fraction of yes decisions, wanting ratings (averaged over all sweet snack and fruit items), and implicit food craving (differences in hunger ratings post-pre) as covariates. Age and total intracranial volume were included to account for the confounding effects of age and individual brain size. We assessed obesity-related gray matter volume differences on a whole-brain level. According to our specific hypothesis of an association between NAcc volume and willingness to exert effort (fraction of yes decisions), as well as between NAcc volume and measures of food craving (wanting ratings and task induced implicit food craving), we utilized a ROI-based approach with a NAcc mask obtained from the Harvard-Oxford Subcortical Structural Atlas within FSL 4.1 to test for related effects. Family-wise Error (FWE) correction for multiple comparisons was applied at the cluster level with a statistical threshold of *p* < 0.05.

## Results

### Demographics, ratings, and questionnaires

Statistical assessment of demographic, rating, and questionnaire data as well as subjects' maximum hand grip forces, mean reaction times and fraction of decisions to exert effort over the whole task are summarized in Table 1. Subject groups did not differ with respect to age and educational background, depressive symptoms, reward or punishment sensitivity, and chronic stress levels. Self-reported body dissatisfaction differed significantly between groups [*F*_(3, 56)_ = 22.62, *p* < 0.001]. *Post-hoc T*-Test revealed that lean men reported lower body dissatisfaction than obese men [*T*_(26)_ = −7.16, *p* < 0.001] and lean women reported lower body dissatisfaction than obese women [*T*_(27)_ = −4.10, *p* < 0.001]. Further, lean men reported lower body dissatisfaction than lean women [*T*_(26)_ = −4.46, *p* < 0.001].

Participants did not significantly differ with respect to hunger prior to task or task-induced implicit food craving (see Table 1). While we found no differences with respect to self-reported liking of the fruit items that entered the task, subjects differed with respect to liking of the sweet snack items [*H*_(3)_ = 8.13, *p* < 0.05]. *Post-hoc* analysis revealed that lean women showed higher liking of sweet snack items than obese men [*H*_(2)_ = 16.6, *p* < 0.01]. All other pairwise comparisons did not survive correction for multiple comparisons. Importantly, all four subject groups showed comparable wanting ratings of the individual sweet snack and fruit items that entered the task (see Table 1).

Maximum hand grip force assessed prior to the task differed between subject groups [*F*_(3, 56)_ = 34.96, *p* < 0.001, Table 1]. *Post-hoc T*-Tests revealed that this was related to gender differences. Both lean and obese men showed higher hand grip force compared with lean women [obese men: *T*_(28)_ = 7.59, *p* < 0.001; lean men: *T*_(26)_ = 6.80, *p* < 0.001] and obese women [obese men: *T*_(27)_ = 7.35, *p* < 0.001; lean men: *T*_(25)_ = 6.56, *p* < 0.001]. In the subsequent task, effort levels were adjusted to individual maximum hand grip force.

### Reaction time and cost-benefit decisions

#### Task-design

Subjects carefully evaluated the tradeoff between costs and benefits. This was reflected in subjects' reaction times by a three-way interaction of effort magnitude, reward magnitude, and fraction of decisions to exert effort (X^2^ = 42.46, *p* < 0.001, Figure 1E). Subjects decided fastest to grip in trials involving low effort (le) and high reward (hr) levels and were slowest in accepting cost-benefit offers in trials with high effort (he) and low reward (lr) magnitude [EMM(le/hr) = 578.61ms, EMM(he/lr) = 621.68ms, *p* < 0.001]. For deciding to reject cost-benefit offers the opposite pattern was observable [EMM(le/hr) = 737.18 ms, EMM(he/lr) = 626.26 ms, *p* < 0.001]. This was mirrored in subjects' decisions, i.e., they more often chose to grip in low compared with high effort trials (X^2^ = 200.74, *b* = 1.56, *p* < 0.001, Figure 1C) and in high compared with low reward trials (X^2^ = 160.44, *b* = 2.33, *p* < 0.001, Figure 1C). As expected, individual wanting ratings of included reward items correlated positively with the amount of exerted effort (*X*^2^ = 17.15, *b* = 0.29, *p* < 0.001) and subjects' willingness to invest effort to receive rewards decreased over time (*X*^2^ = 67.17, *p* < 0.001, Figure 1B). Comparing reward categories, we observed that subjects significantly faster decided in trials that yielded money than fruit items (*X*^2^ = 5.68, *b* = −17.6, *p* < 0.05). Subjects also more often chose to grip for money than for sweet snacks (*X*^2^ = 16.47, *b* = 1.14, *p* < 0.001, Figure 1D) or fruit pieces (*X*^2^ = 5.1, *b* = 0.72, *p* < 0.05, Figure 1D).

Whether reward or effort demand was depicted first also influenced subjects' task performance. They decided faster whether to grip or not in trials in which reward stimuli were displayed before effort demands were shown (X^2^ = 41.69, *b* = −29.63, *p* < 0.001). In these trials, subjects were also more likely to invest effort than in trials, in which effort levels were shown first (*X*^2^ = 6.97, *b* = 0.1, *p* < 0.01). Taken together, these results support the ecological validity of our approach.

#### Obesity- and gender-related effects

Throughout the task, lean subjects responded faster than obese participants (*X*^2^ = 4.67, *b* = −44.05, *p* < 0.05, Table 1). We found no association of obesity and general amount of exerted effort throughout the task (Table 1). However, we observed a significant obesity by gender interaction (*X*^2^ = 5.12, *p* < 0.05): Obese men less likely invested effort than lean men [EMM(lm) = 0.81, EMM(om) = 0.63, *p* < 0.01, Table 1]. This obesity-related difference was not observable in women [EMM(lw) = 0.71, EMM(ow) = 0.75, *p* = 0.59].

**Figure 2:**
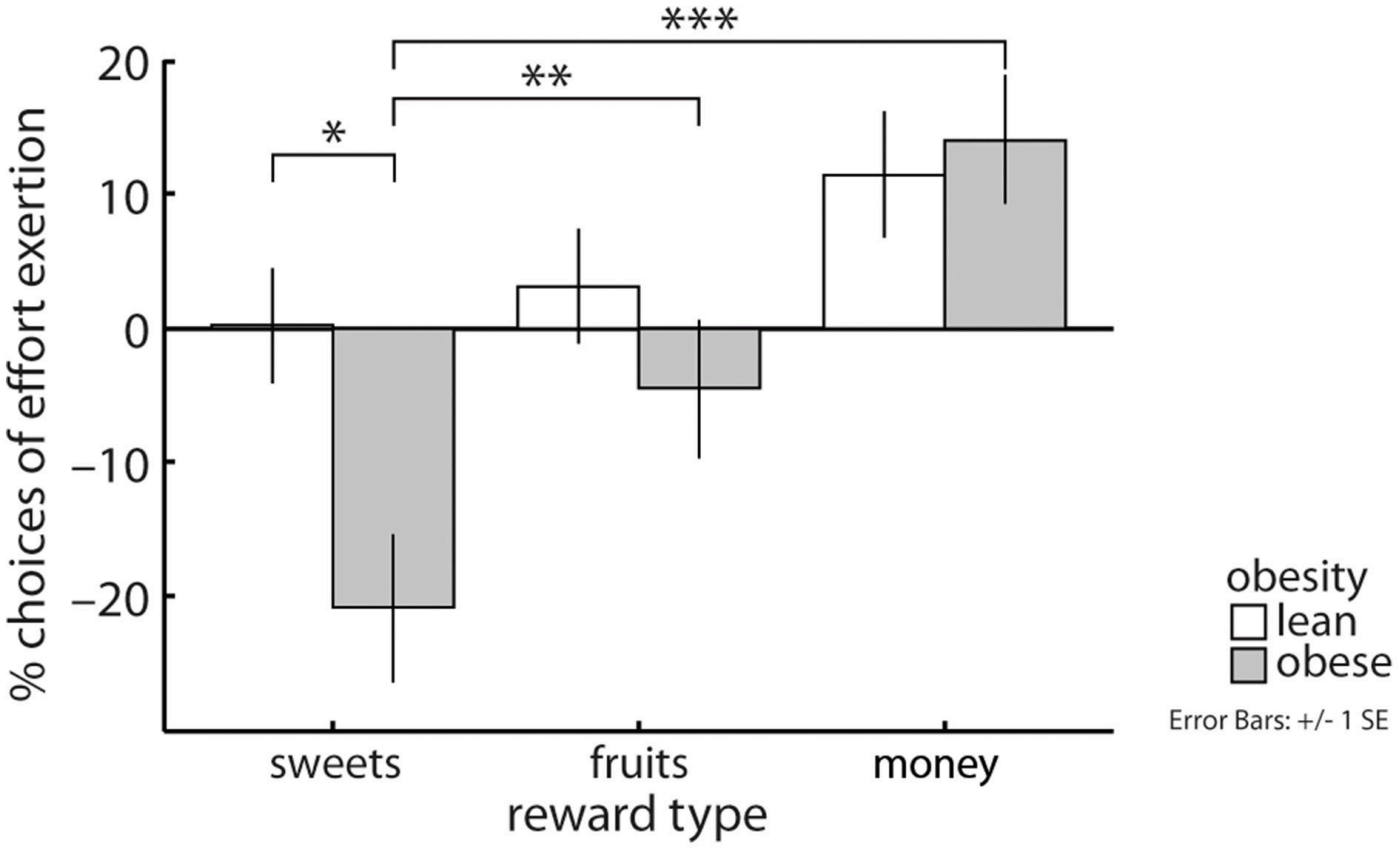
An interaction between reward category and obesity revealed that obese compared with lean subjects less often chose to grip for sweet high-caloric snacks, but performed similarly with respect to fruits and money as rewards. Obese subjects also more often decided to grip for money and for fruits than for sweets, this effect was not apparent in lean subjects. Depicted values are corrected for factors and covariates within the respective GEE model. Asterisks indicate significance within the respective GEE model reported in the Results Section.

As hypothesized, our data showed an obesity by reward category interaction (*X*^2^ = 7.92, *p* < 0.05, Figure 2): Obese compared with lean subjects were less likely to grip on trials yielding sweet snacks [EMM(l) = 0.71, EMM(o) = 0.5, *p* < 0.05] but not on trials involving fruit pieces or money as a reward. Obese subjects more often decided to grip for money and for fruits than for sweet snacks [EMM(mo) = 0.85, EMM(sw) = 0.5, *p* < 0.001; EMM(fr) = 0.66, *p* < 0.01]. Lean subjects equally often decided to grip regarding the three reward categories.

Reaction time analysis further revealed a significant interaction of gender by reward category (*X*^2^ = 9.05, *p* < 0.05, Figure 1F). Men decided faster in trials that yielded monetary reward (mo) compared to fruit (fr) and sweet snack (sw) trials [EMM(mo) = 590.21 ms, EMM(fr) = 631.38, *p* < 0.01; EMM(sw) = 625.79, *p* < 0.01], but no such differentiation was found in women. With respect to subjects' decisions, we found a significant effort level by gender interaction [X^2^ = 5.32, *p* < 0.05]. Specifically, women were more sensitive to an increase in effort demands than men, i.e., women were more likely to invest effort in low effort trials than men, but men more often decided to exert effort in high effort trials than women [low effort: EMM(w) = 0.88, EMM(m) = 0.85; high effort: EMM(w) = 0.51, EMM(m) = 0.56; *X*^2^ = 186.86, *p* < 0.001]. We did not find significant interactions of trial block by obesity, trial block by gender or obesity by gender and reward category.

#### Influence of age, chronic stress, reward/punishment sensitivity, and body dissatisfaction

**Figure 3:**
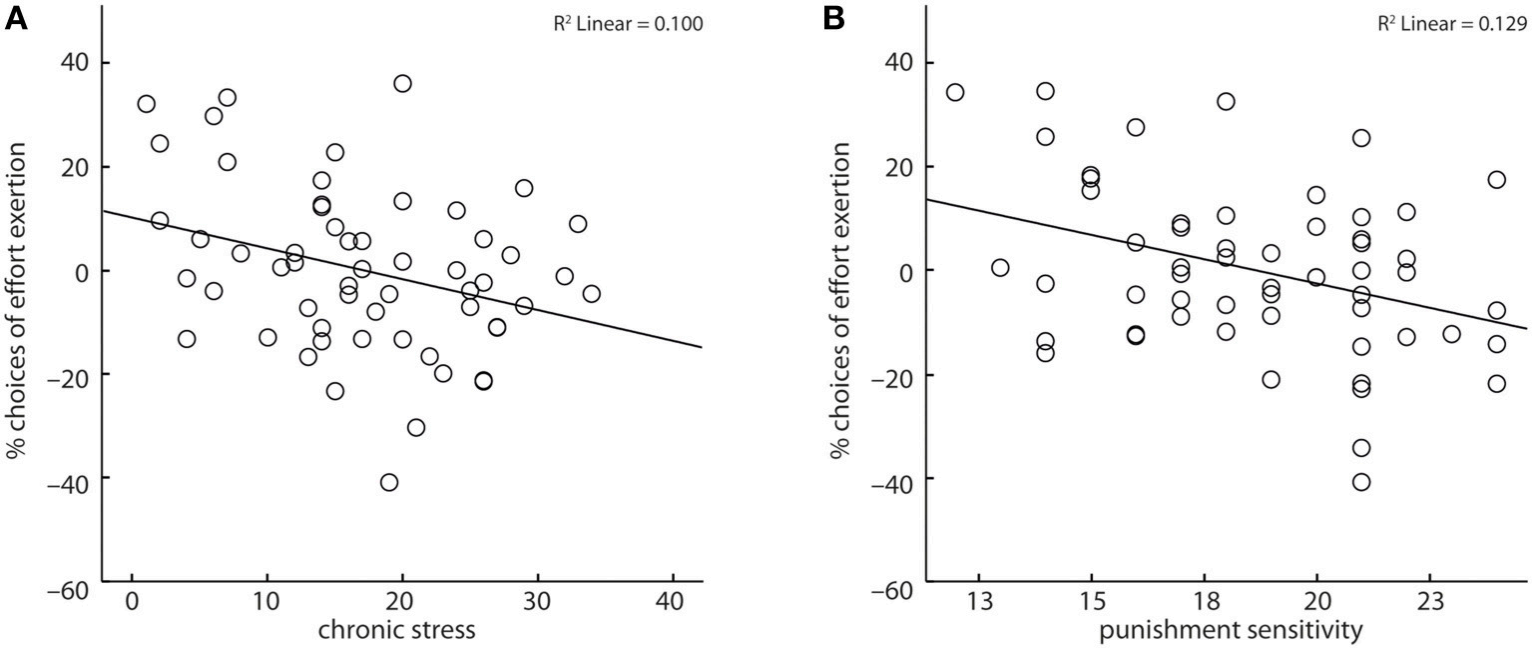
Chronic stress **(A)** and punishment sensitivity **(B)** correlated negatively with the likelihood of choosing to exert effort. Depicted values are corrected for factors and covariates within the respective GEE model.

Across all subjects, reaction times marginally increased (*X*^2^ = 3.53, *b* = 3.02, *p* < 0.06) and subjects' amount of expended effort decreased with increasing chronic stress (*X*^2^ = 5.47, *b* = −0.03, *p* < 0.05, Figure 3A). Further, subjects' punishment sensitivity correlated negatively (*X*^2^ = 11.34, *b* = −0.14, *p* < 0.001, Figure 3B) with the fraction of decisions to invest effort. Age and reward sensitivity had no significant impact on subjects' decisions, though with increasing age reaction times increased (*X*^2^ = 6.03, *b* = 7.57, *p* < 0.05).

**Figure 4:**
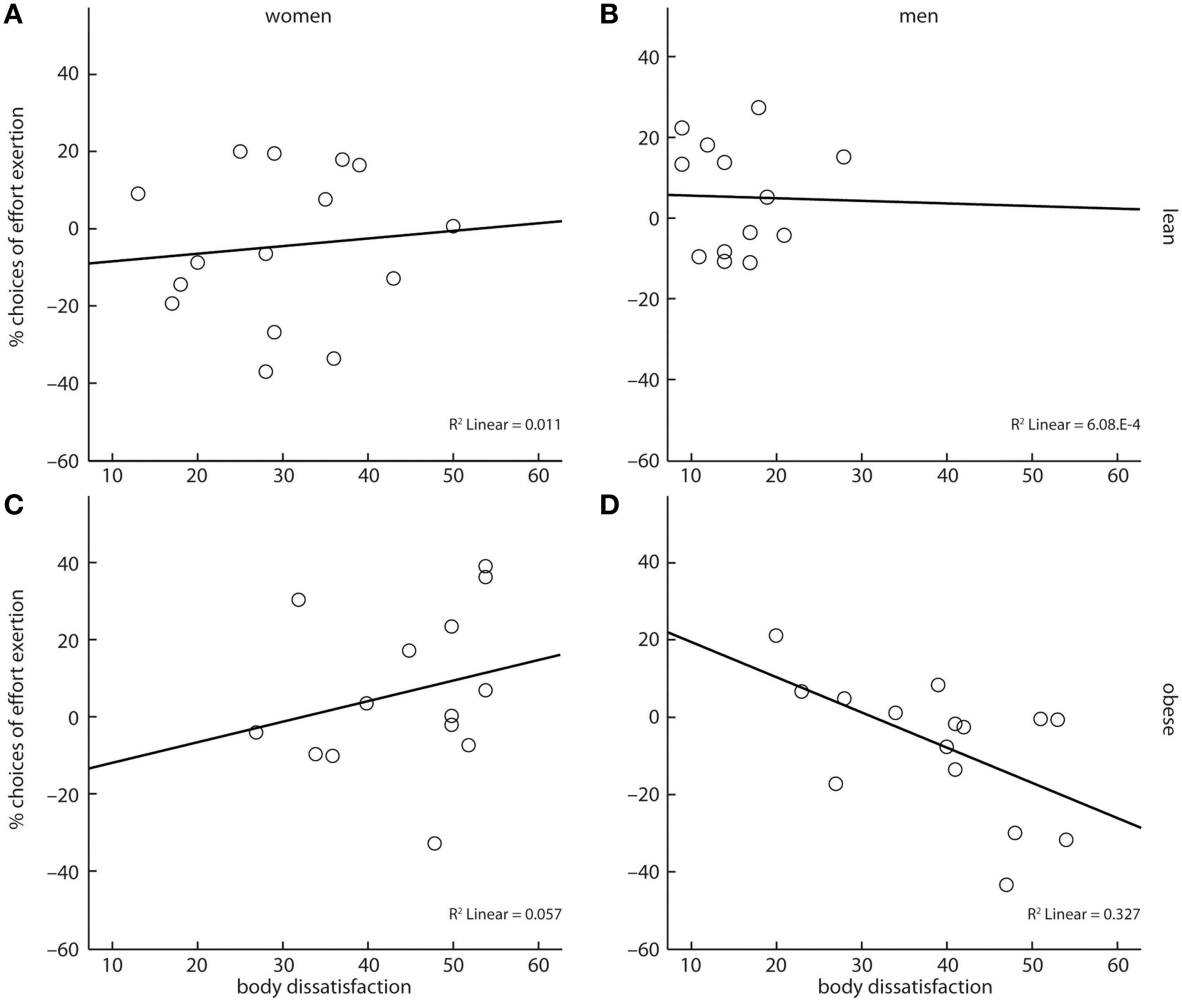
A four-way interaction between reward category, obesity, gender, and body dissatisfaction showed that obese men's cost-benefit decisions regarding sweet snacks were negatively correlated with their self-reported body dissatisfaction **(D)**. No such association was observed for lean women **(A)**, lean men **(B)**, and obese women **(C)**. Depicted values are corrected for factors and covariates within the respective GEE model. Asterisks depict significance within the GEE model.

Following our hypothesis, we finally investigated if body dissatisfaction had an impact on cost-benefit decisions. We assessed a four-way interaction of reward category, obesity, gender, and reported body dissatisfaction. This interaction was significant (*X*^2^ = 37.33, *p* < 0.001, Figures 4A–D). However, contrary to our hypothesis, parameter estimates of the model showed that body dissatisfaction negatively correlated with the likelihood to grip for sweet snacks in obese men (*X*^2^ = 5.48, *b* = −0.05, *p* < 0.05, Figure 4A), but not in obese women (*X*^2^ = 1.44, *b* = 0.05, *p* = 0.23, Figure 4B).

#### Structural MRI

**Figure 5:**
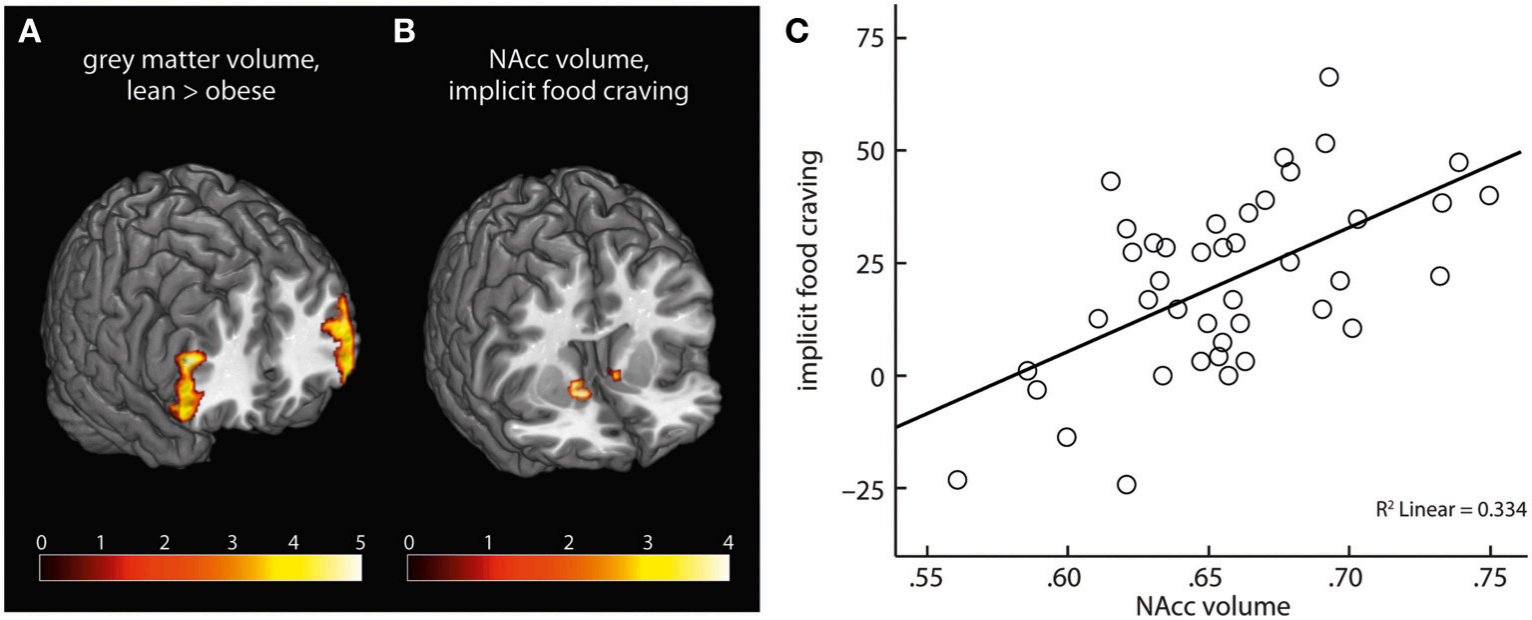
Obese subjects had lower gray matter volume in bilateral PFC compared with lean participants **(A)**. NAcc volume positively correlated with severity of implicit food craving **(B,C)**.

On a whole-brain level, obese compared with lean subjects had lower gray matter volume in bilateral clusters of ventrolateral PFC, comprising inferior frontal gyrus (Table 2, Figure 5A). We found no positive association of obesity and gray matter volume.

**Table 2:** **Results from the VBM analysis in a subsample of 42 subjects**.

	**MNI-coordinates (peak voxel)**	**Number of voxels**	**Z-Score**
**LEAN** > **OBESE SUBJECTS (WHOLE-BRAIN)**			
Right inferior frontal gyrus	54, 39, 9	1134	4.43
Left inferior frontal gyrus	–50, 30, 18	2091	4.73
**IMPLICIT FOOD CRAVING (ROI-BASED)**			
Right NAcc	10, 15, −11	158	3.64
Left NAcc	–3, 14, −2	30	3.63

*Lean (23) compared with obese subjects had higher gray matter volume in bilateral inferior frontal gyrus. NAcc volume correlated positively with implicit food craving severity*.

ROI analysis of NAcc gray matter volume yielded a significant positive correlation of implicit food craving and bilateral NAcc volume (Table 2, Figures 5B,C). We found no significant association of NAcc volume and subjects' willingness to exert effort, or explicit wanting ratings.

## Discussion

Here we show that the trade-off between costs in terms of physical effort and food reward in obese subjects may be more complex than expected up to date (Epstein et al., 2007; Giesen et al., 2010). Our data demonstrate for the first time that obese compared with lean subjects may be less willing to invest physical effort for high-caloric food reward in particular. Importantly, in a recent study that utilized button presses to obtain a reward, we observed that obese men were less sensitive to changes in motivational value of snack food, as induced via a devaluation procedure, than lean men (Horstmann et al., 2015a). This indicates that the obesity-related difference observed here is specific for physical effort, emphasizing the importance of taking into account physical effort as a potential target for therapeutic interventions and changing every day food choices of obese subjects possibly via increasing effort barriers, e.g., by rearrangement of food assortments in cafeterias and supermarkets. Two recent studies in humans (Epstein et al., 2007; Giesen et al., 2010) revealed contradictory results to our finding, which most likely reflects methodological differences. We used three distinct reward categories and, in contrast to previous studies that employed button presses, assessed physical effort via a handgrip dynamometer which has proven to be a reliable tool to capture physical exertion (e.g., Treadway et al., 2009; Wardle et al., 2012). Notably, findings in rodents related to cost-benefit decision-making alterations in obesity models are also diverse. While some studies show an increased motivation to work for high-caloric sweet food in rodent models of obesity (la Fleur et al., 2007; Hajnal et al., 2008; Narayanaswami et al., 2013), there is also growing evidence for a decreased willingness to exert effort for food high in fat and sugar (Davis et al., 2008; Shin et al., 2011; Harb and Almeida, 2014). This reduced motivation to work for food reward may partly be associated with an attenuated dopamine metabolism, possibly reflecting adaptive processes such as heightened DA level within NAcc as a result from a prolonged energy-dense diet (Davis et al., 2008). Studies in humans show reduced striatal D2/D3 receptor availability in obese compared with lean subjects that may relate to heightened tonic DA as well (Horstmann et al., 2015b). Following this, our findings may hint at similar associations between disturbances in dopaminergic function and reduced motivation to exert effort for high-caloric food in obese individuals.

The observed obesity-associated difference in cost-benefit evaluation was modulated by self-reported body dissatisfaction and gender. In obese men, but not in obese women, the likelihood of choosing to invest physical effort for high-caloric sweet snacks was negatively correlated with body image discontent. We expected to observe an impact of body dissatisfaction on cost-benefit decisions rather in obese women than in obese men, due to the supposed greater burden of stigmatization in obese women (Gray et al., 2011; Forrester-Knauss and Zemp Stutz, 2012; Forste and Moore, 2012). Our opposing observation hints at a greater awareness and impact of weight discrimination on behavior in men than expected to date. In accordance, Lieberman et al. (2012) recently reported that men showed greater negative attitudes toward obesity than women. Further, they found that BMI positively correlated with the strength of negative attitudes toward obesity in men but not in women. The higher the BMI of their male participants was, the more they were concerned with the fear of getting obese. In addition, frequency of consuming fast-food is higher in men than in women and correlates positively with BMI (Dave et al., 2009; Anderson et al., 2011). A prolonged period of consuming convenience products may foster the association between palatable food and low effort demands. Further, prolonged consumption of palatable food can lead to a decrease in liking of these food items (Clark et al., 2010).

The modulating effect of gender on the observed obesity-associated differences in cost-benefit evaluation may also be related to differences in dopaminergic tone within the fronto-striatal pathway (e.g., Haaxma et al., 2007). Estrogen level was shown to impact measures of dopamine-related cognitive performance and inhibitory control (Colzato et al., 2010; Jacobs and D'Esposito, 2011; Silverman et al., 2011; Hampson and Morley, 2013). In rodents, modification of estradiol levels was previously related to alterations in cost-benefit decision-making (Uban et al., 2012).

Subjects choose to invest effort to receive a certain reward if their subjective motivational value of the reward item exceeds the respective effort costs. Accordingly, individual wanting ratings correlated positively with subjects' choices. Notably, we also tested whether controlling for liking of the food items would change our obesity-associated results. This was not the case. Thus, behavior in our task was driven by the current motivational value of the rewards. Literature on obesity-associated differences with respect to wanting and liking of sweet snack food is still inconsistent. Despite studies showing a positive relationship between the reinforcing value of snack food and body weight (Ouwehand and de Ridder, 2008; Goldfield et al., 2011; Ochner et al., 2012), there is also evidence of a possible negative association (Cox et al., 1998; Gearhardt et al., 2014). Consumption of high-caloric food over a prolonged period can decrease liking of energy-dense food (Clark et al., 2010; Vucetic et al., 2011).

Women were more sensitive to increases in physical effort demands than men. This is in accordance with previous findings, indicating that men rated perceived exertion lower than women (Skatrud-Mickelson et al., 2011). A possible contribution to this gender-associated difference may arise from motivation intensity theory, indicating that men are more likely to be motivated by performance incentives (Barreto et al., 2012). Further, Perciavalle et al. (2010) showed that motor cortex in women is more sensitive to increases in circulating blood lactate levels than men's motor cortex, hinting at sensitivity differences of physical exertion on a neural level.

Subjects' punishment sensitivity negatively correlated with their willingness to exert effort. This indicates that in our task design, effort exertion via pressing a handgrip device was perceived as physically demanding by the participants. This is an important aspect regarding the comparability with studies that used button presses as a measure of effort costs and that reported different results with respect to obesity (e.g., Giesen et al., 2010). Punishment sensitivity is associated with dopaminergic tone, specifically within the right frontal cortex and striatum (e.g., Maril et al., 2013). On a neural level, individual tonic dopamine level differences within the right fronto-striatal pathway may thus have contributed to sensitivity differences with respect to effort demands in our task.

Notably, self-reported chronic stress levels were negatively associated with subjects' likelihood of choosing to grip for rewards. This novel finding in humans is in accordance with a recent observation in rodents (Shafiei et al., 2012) that acute stress diminished the preference of rats to exert high effort levels to receive rewards. Stress is known to affect dopaminergic transmission within the fronto-striatal circuitry (Roth et al., 1988; Abercrombie et al., 1989; Davis et al., 1994; Latagliata et al., 2014). Since the same dopaminergic pathways are involved in processing effort-related information during cost-benefit decision-making (Walton et al., 2003; Schweimer and Hauber, 2006; Salamone et al., 2007; Salamone and Correa, 2012), stress may alter cost-benefit decision-making via modifying dopamine transmission in fronto-striatal dopaminergic target regions.

With respect to brain structure, we found lower gray matter volume in bilateral PFC comprising large parts of inferior frontal gyrus in obese compared with lean participants in a subsample of 42 participants. IFG is involved in inhibitory control mechanisms to guide behavior in a goal-directed manner among a variety of other processes (e.g., Aron et al., 2004; Swann et al., 2009). Hypo-functioning and lower gray matter volume within IFG have been related to obesity and disordered eating before (Batterink et al., 2010; Sweet et al., 2012; Balodis et al., 2013; Brooks et al., 2013). Regarding the motivation to overcome physical effort, Treadway et al. (2012) found that dopamine responsivity within IFG among other regions positively predicted willingness to exert effort in trials entailing high effort demands but low reward probabilities. In line, Massar et al. (2015) recently showed that IFG is critical for coding effortful demands. In contrast to our recent findings (Horstmann et al., 2011), we did not observe a positive association of obesity and gray matter volume in reward related areas or a modulatory effect of gender on gray matter volumetric differences regarding obesity. This may be related to the relatively small sample size compared with the former study.

As hypothesized, gray matter volume of NAcc positively correlated with an implicit measure of task-induced craving for food, as assessed by differences in subjects' hunger ratings before and after task execution. This is in line with recent findings that showed NAcc activity to correlate with craving severity in smokers (Kober et al., 2010). As we only assessed brain structure in this study, the next crucial step is to assess brain function during cost-benefit decision-making, to follow up on this finding. Associations between local gray matter volume and BOLD activation are complex, and dependent on the specific region of interest and the respective task among a multitude of other factors. Besides no direct associations (Guo et al., 2015) there are also studies showing a positive (Kalpouzos et al., 2012; Pujol et al., 2013) but also a negative correlation between local gray matter volume and BOLD response (Johnson et al., 2000; Bartrés-Faz et al., 2009; Kalpouzos et al., 2012).

Subjects' reaction times were modulated by reward and effort magnitude in a differential manner for yes and no decisions. Thus, subjects carefully evaluated reward and effort information and integrated both to form a decision. In line with this observation, Basten et al. (2010) recently proposed that cost-benefit decisions were established in an analogous way to perceptual decisions, i.e., the brain weighs costs and benefits by accumulating the difference signal of both on a neural level until a decision threshold is reached. Further, participants with obesity responded slower throughout the task than lean subjects. This is a common finding with respect to both simple (e.g., Khode et al., 2012; Gentier et al., 2013; Hagger-Johnson et al., 2014) and cognitive more demanding tasks (e.g., Nederkoorn et al., 2012; Gentier et al., 2013; Kamijo et al., 2014). In addition, men decided faster in trials involving monetary compared to food reward. This is congruent with recent observations that men but not women responded faster in a reaction time task involving monetary compared with social reward and that men revealed a differential BOLD activation pattern during anticipation of the distinct reward types (Spreckelmeyer et al., 2009).

In conclusion, our novel findings shed new light on obesity-related alterations in cost-benefit decision-making. Former findings that obese individuals may be willing to work harder for high-caloric food are challenged. Moreover, obese men seem to be more affected by concerns about body shape and possibly by related stigmatization than previously expected. This is an important issue for therapeutic strategies aiming at weight reduction and reducing stigmata in obese men. Further, increasing effort barriers for high-caloric food in food and eating environments (e.g., cafeterias, supermarkets), for example, by repositioning food assortments, may prove as a powerful tool to influence eating behavior. Additionally, therapeutic interventions aiming at altering psycho-social burdens such as stress may help to positively influence everyday-life effortful decisions and thereby reduce positive energy-balances in obese individuals.

## Author contributions

DM, AH, BP, AV, and JN conceived and designed the experiment, DM acquired data, DM, AH, JN performed data analysis. DM, AH, BP, AV, and JN prepared and revised the manuscript. JN supervised the study.

## Funding

This work was supported by the Federal Ministry of Education and Research (BMBF), Germany (FKZ: 01EO1001), the German Research Foundation (DFG-SFB1052), the FAZIT-STIFTUNG (FAZIT-STIFTUNG Gemeinnützige Verlagsgesellschaft mbH), and the Free State of Saxony (Landesstipendium).

### Conflict of interest statement

The authors declare that the research was conducted in the absence of any commercial or financial relationships that could be construed as a potential conflict of interest.
